# GLSNN Network: A Multi-Scale Spatiotemporal Prediction Model for Urban Traffic Flow

**DOI:** 10.3390/s22228880

**Published:** 2022-11-17

**Authors:** Benhe Cai, Yanhui Wang, Chong Huang, Jiahao Liu, Wenxin Teng

**Affiliations:** 1 Key Laboratory of 3-Dimensional Information Acquisition and Application, Ministry of Education, Capital Normal University, Beijing 100048, China; 2 State Key Laboratory of Resources and Environmental Information System, Institute of Geographic Sciences and Natural Resources Research, Chinese Academy of Sciences, Beijing 100101, China

**Keywords:** GLSNN model, spatiotemporal data fusion, multi-scale, traffic flow prediction, spatiotemporal dependence

## Abstract

Traffic flow prediction is a key issue in intelligent transportation systems. The growing trend in data disclosure has created more potential sources for the input for predictive models, posing new challenges to the prediction of traffic flow in the era of big data. In this study, the prediction of urban traffic flow was regarded as a spatiotemporal prediction problem, focusing on the traffic speed. A Graph LSTM (Long Short-Term Memory) Spatiotemporal Neural Network (GLSNN) model was constructed to perform a multi-scale spatiotemporal fusion prediction based on the multi-source input data. The GLSNN model consists of three parts: MS-LSTM, LZ-GCN, and LSTM-GRU. We used the MS-LSTM module to scale the traffic timing data, and then used the LZ-GCN network and the LSTM-GRU network to capture both the time and space dependencies. The model was tested on a real traffic dataset, and the experiment results verified the superior performance of the GLSNN model on both a high-precision and multi-scale prediction of urban traffic flow.

## 1. Introduction

Intelligent transportation systems (ITS) are the main means to solve the current urban traffic problems, and the prediction of traffic flow is the key technical link in the system. Traffic prediction has a decisive role in whether the system can provide accurate and timely service responses. The traditional traffic flow prediction algorithms only forecast from historical and current traffic information, but traffic conditions can be affected by the road network, peak periods, and other factors. Therefore, a more efficient traffic flow forecasting system is needed to improve the efficiency of urban traffic operations. 

Data fusion technology allows data from different sources to be combined in one model, including flow data from different sensors, or a free flow input. This fusion, however, is considered to be a major challenge for the accurate prediction of the flow of urban traffic. Combining specific degrees of spatiotemporal complexity and exogenous models is most likely to be an optimal choice for improving the accuracy. After extending the prediction methods of a multi-source data fusion, researchers have also introduced deep learning neural networks from the perspective of deepening mathematical analysis models, but most studies only make time-series predictions for each road independently and do not consider the traffic state spread problem generated by the topological relationship between the roads. In addition, due to the limitations of the model performance, most of the existing research has used a short-term input, which cannot take into account the change in the traffic flow rules caused by a seasonal transformation. Therefore, this paper constructs a multi-scale spatiotemporal prediction model from the perspective of the traffic flow prediction. The model learns the large-scale road network topology in spatial units of different scales, and a scale segmentation module is established to receive input from multi-scale time-series big data. Finally, the model is able to predict traffic flow with a high degree of precision over a long time span and a wide area. 

## 2. Related Work

Traffic flow prediction research originally adopted the traditional parameterization method, which mainly includes two methods: prediction using a time series and prediction using a Kalman filter. The time-series method predicts by fitting parameters, and the most widely used is the autoregressive integrated moving average model (ARIMA) [1]. The Kalman filter method is based on the traffic state of the previous moment to predict future traffic conditions. However, because the traffic flow itself is nonlinear and uncertain, the robustness of these parametric method models is very poor. Since the traffic flow data are random, non-parametric methods have been generated, the most common of which are machine learning methods, such as support vector regression (SVR), k-nearest neighbor (KNN), a support vector machine (SVM), and artificial neural networks (ANN). 

With the deployment of sensors and the improved temporal resolution of traffic flow data, it is increasingly difficult to use existing methods to deal with the challenges of increasing data. Moreover, modern ITS requires a deeper exploration of potential traffic information and its internal connections. Therefore, there is an urgent need for new big data mining analysis techniques that can handle deeper levels. Because of this deepening of the applications of artificial intelligence in various fields, researchers began to use deep learning methods to solve the traffic flow prediction problem [2,3,4]. Koesdwiady et al. [5] proposed a traffic data architecture based on deep belief networks (DBN). Lv et al. [6] explored the use of a stacked autoencoder (SAE) for the prediction of the traffic flow and successfully implemented a neural network. Laptev et al. [7] used recurrent neural networks (RNN) to predict time-series data to effectively learn high-dimensional features in traffic data and improve the prediction performance. Zhang et al. [8] converted the road network into a two-dimensional grid and applied convolutional neural networks (CNN) to predict the traffic flow. Still, none of these studies achieved an ideal prediction accuracy. Then, as variants of RNN such as long short-term memory networks (LSTM) and gated recurrent units (GRU) were found to solve the problem of gradient disappearance in RNN, more research began to use LSTM or GRU to learn the time dependence of traffic data [9,10]. However, these models do not capture time and space dependencies at the same time, which leads to changes in the traffic state not being captured and propagated. Therefore, there is a need for an algorithm that can simultaneously capture the time dependence and spatial dependence of traffic data features in the case of complex urban road networks and topology.

Based on this demand, researchers began to use a variety of graph neural networks and variants of cyclic neural networks to combine training and prediction. For example, Ma et al. [11,12] attempted to use convolutional neural networks (CNN) and the LSTM model fusion for predictions, and the experiment results significantly beat the single-model independent prediction. However, the CNN-based method is only suitable for spatial relationships using Euclidean distance and cannot learn the urban road network structure well. This will cause large fluctuations in the prediction accuracy. A new graph convolutional network (GCN) was developed and used for a spatial convolution in non-Euclidean space. For example, Yuan et al. and Li et al. [13,14] used a combination of the GCN and RNN to predict the traffic status. The study proved the feasibility of the GCN for the prediction of urban road network traffic flow, but the prediction still did not exceed the accuracy of the CNN method. Later, Yu et al. [15] proposed a spatiotemporal graph convolutional network that uses the GCN to graphically model the research space and convolute the time axis to simulate the time and space dependence and make comprehensive predictions. Nonetheless, it achieved a more accurate prediction in only a small part of the study area. Zhao et al. [13] used GCN to capture road topology and use gated recurrent units (GRU) to capture dynamic changes in the traffic data. The method beat the baseline method, but it neglected the need for a higher prediction accuracy in urban traffic arteries when capturing complex urban topology. 

In summary, the above-mentioned fusion prediction models focus on the prediction of the traffic flow in a short time span in a small area, and few studies have been committed to the prediction of large-scale networks. There has also been a lack of comprehensive parameters for modeling urban arterial road traffic, resulting in a low prediction accuracy. Therefore, we present a traffic flow prediction model that can capture both the time dependence and spatial dependence. The result is a spatiotemporal prediction of the urban traffic flow at multiple input and output scales. We designed three modules—time-series data scale segmentation, road network spatial topology learning, and time-series data training prediction—and integrated the GLSNN model for the prediction of the traffic flow.

## 3. Methodology

In this paper, the purpose of traffic forecasting is to predict the traffic flow conditions for a period of time in the future based on historical road traffic flow data and urban road network topology [16]. Traffic flow data are a broad concept and can be the traffic’s speed, volume, or density. According to the current literature on the prediction of the traffic flow [17,18], we used the traffic’s speed as the result parameter of the traffic flow prediction model.

**Figure 1:**
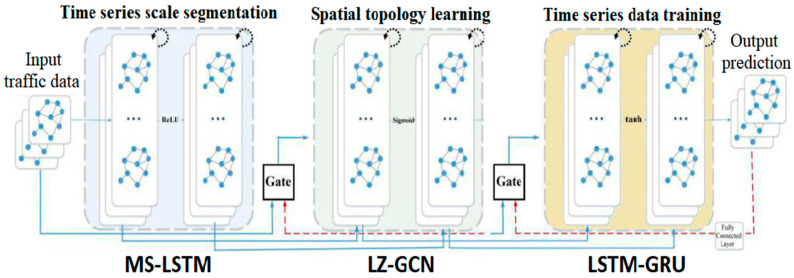
Architecture of GLSNN.

As shown in Figure 1, our GLSNN model is divided into three parts: MS-LSTM, LZ-GCN, and LSTM-GRU. First, the historical traffic speed data are used as the input to “feed” the MS-LSTM module for a scale division of an ultra-long time series, and the LZ-GCN module is used to learn the spatial weight matrix of the topological structure between the roads. Finally, the multi-scale time series with spatial features are transmitted to the LSTM-GRU module. The dynamic transfer between cells is carried out through the forget gate and combined with non-traffic factors for the prediction. Finally, the results are output through the fully connected layer. Our model structure is as follows.

### 3.1. Scale Segmentation of Huge Traffic Flow Data Based on MS-LSTM

The first part of the model is the MS-LSTM module (multi-scale LSTM), which is used to scale the huge traffic flow data to better use the time-series data to train the prediction model. 

In the previous study of the prediction of the traffic flow, the traffic time-series data for 1–3 months was selected as the dataset for training; however, such an approach does not apply to the prediction of a date different from the season in which the training set is located, and inputting data for the whole year may result in a slow calculation or parameter overflow. In order to increase the time span of the input data and achieve an accurate output of different spatial ranges and time step prediction results, we explore how to make the prediction model simultaneously learn the different rules of a traffic flow change. This paper adopts a method of integrating a tree structure into an LSTM network to realize a scale segmentation of the time-series data. It can not only learn hierarchical structure information but also improve the training efficiency of the large-capacity model.

In order to learn a greater span of massive traffic flow information at various scales, the encoding of MS-LSTM was designed to distinguish between high- and low-level information because in such a sorted LSTM network of neurons, higher-level information is more easily preserved through the forget gate, while lower-level information is more easily forgotten in its corresponding interval. Traffic flows have a hierarchical structure, and smaller units (such as hourly or every 5 min) are nested in larger units (such as daily), forming a strict hierarchy. Although the LSTM network can use different neurons to track information at different time scales, the standard architecture does not impose this strict hierarchy. At the end of the day, the traffic flow tends to zero, and the data at this time has lost its statistical significance [19]. Therefore, the daily unit is turned off at this time, and the hourly unit and the 5 min unit are also reset. However, the full hourly and 5 min models of the day learned by the unit are retained by the unit’s forget gate each day. In addition, the hour unit model for daily commutes has peaks and troughs and the day unit model for the workdays and weekends is also intensively trained through the gating mechanisms. This paper strengthens the model’s capability to differentiate and generalize the multi-scale hierarchical structure by sorting the cells. The nested information implicit in the input sequence can be obtained by recursing the data after the segmentation.

**Figure 2:**
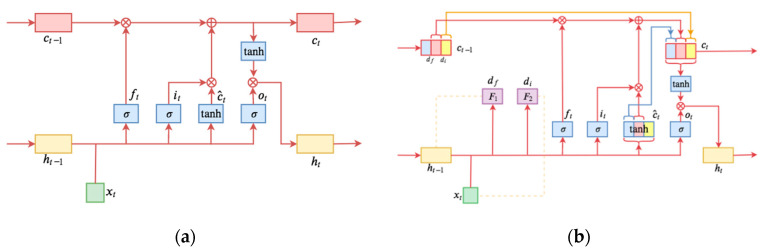
Comparison of LSTM and MS-LSTM: (**a**) Standard LSTM; (**b**) MS-LSTM.

As shown in the structural diagram in Figure 2, the core of MS-LSTM is the definition of $f_{t}$ and $i_{t}$, and $f_{t}$, $i_{t}$, and $o_{t}$ are the three single-layer fully connected models. The input is the historical information $h_{t-1}$ and current information $x_{t}$, activated by the sigmoid. The result of the sigmoid is between 0 and 1, and there are three “gates”: the forget gate, input gate, and output gate. In a common neural network, neurons are usually disordered. If the positions of all the vectors involved in the LSTM operation are re-scrambled in the same way, the weight order is also shuffled accordingly, and then the output result can be just the reordering of the original vectors. $h_{t-1}$ indicates the hidden state at time t−1; $x_{t}$ is traffic information indicating time t; $r_{t}$ is a reset gate used to control the extent to which the status information is ignored at a previous time; $u_{t}$ is an update gate for controlling the extent to which the previous state’s information entered the current state; $c_{t}$ is the memory content stored at time t; and $h_{t}$ is the output state at time t. The MS-LSTM obtains the traffic state at time t by taking the hidden state of time t−1 and the current traffic information as the inputs. When capturing the traffic information at the current time, the model retains the trend of the historical traffic information and has the capability to capture the time dependence. This self-learning hierarchy can also reduce the error of some special values in the traffic flow by using velocity data because at some point, the speed value collected by the detector is accidental and does not fully represent a short period of time. At the same time, fewer avoidable error values can also reduce the over-fitting of the neural network, thereby increasing the accuracy of the prediction.

### 3.2. Acquisition and Learning of Multi-Scale Spatial Dependencies of Urban Complex Road Networks Based on the LZ-GCN Network

**Figure 3:**
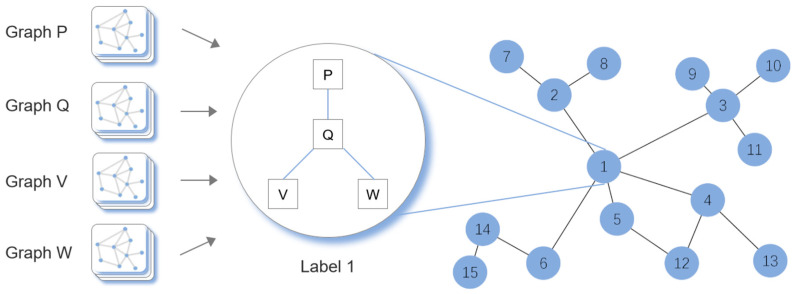
Diagram of LZ-GCN; the numbered circles indicate labels.

The second part of the model is the LZ-GCN (Link Zone Graph Convolutional Network) module. In order to more accurately predict the city road network traffic flow, we built the LZ-GCN network to better capture and learn the complex multi-scale spatial dependence of the urban road network (Figure 3), so that it can be integrated with the LSTM-GRU module.

Whether the spatial weight matrix can be constructed according to the topology between the roads and the actual situation of the urban road network is the key to obtaining the spatial dependence. More and more researchers are experimenting with variants of a CNN that can handle arbitrary graphical structure data, such as the GCN used for learning and training road topology [13]. Although it can overcome the shortcomings of a CNN that can only be used in regular grids, the complex spatial features of urban road networks need to learn their spatial weight matrix more accurately. Due to the special structure of urban road networks, most of the existing traffic flow predictions are only based on the “road segment” to predict the traffic speed. However, it only uses the part between the intersections as the spatial sampling unit of the traffic speed, thus an adequate spatial meshing cannot be achieved for relatively long road segments and secondary intersections which are lacking a weight judgment. In order to achieve a more detailed capture of the urban road network spatial correlation, it is necessary to comprehensively consider the local connectivity and traffic flow delivery mode, so that the nodes, road segments, and areas of the road network can be divided to more accurately simulate the spatial changes in the traffic states, and thus improve the prediction accuracy.

In this paper, the diffusion process of the traffic flow is expressed by means of directed graphs with embodied structural features. The traffic state spreads to the upstream mainly due to a congestion or a slow driving transmission to the upstream road section; the way the traffic state spreads to the downstream is affected by factors such as the driver’s driving habits or the nature of special vehicles. In addition, traffic state diffusion can only be transmitted between adjacent road nodes. Therefore, the state transition matrix should be constructed for the accessibility of a transmission and diffusion between the adjacent nodes and non-adjacent neighboring nodes. In order to better determine the weight of the traffic state diffusion, we use a further fine-grained link to construct the road network space weight matrix and split and classify some special road segments to solve the problem of a delay in the spread of traffic conditions caused by long sections and the problem of an excessive dispersion of weights in secondary intersections. In addition, we constructed a coarse-grained spatial matrix using a “Zone” as the unit in order to improve the prediction accuracy of the relatively unpopular areas with less traffic flow data from the perspective of the urban network prediction. Through the clustering method of similar traffic status sections in the neighborhood, the diffusion weight of the traffic state was more accurately transmitted to the area with less traffic flow data, so as to improve the prediction accuracy of the whole network.

LZ-GCN refers to a graph convolutional neural network’s implementation based on two different spatial scales of Link and Zone. In this paper, we input the graph structure of the road network in the form of a Link and Zone for spatial dependence learning. Then, we abstracted the traffic speed as the signal of the graph node and extracted the graph structure data layer by layer through a graph convolution operation. We used multiple nesting levels for nodes by making multiple changes to the collected signal information from nearby nodes. Finally, we passed the final node nesting layer to the classifier for a node attribute assignment. In this way, our model learned spatial dependence without causing a loss of the urban road network’s topology connectivity and global reachability. By setting the start distance and the end distance, the weaker correlation areas were selected, and the weight of the data output was reduced to reduce the accuracy of the less reliable data to the target area prediction. Furthermore, the predictive model used the learned features to achieve a more reasonable node weight distribution and capture the upstream and downstream signals through the inherent bidirectional feedback characteristic of the graph convolutional network. Finally, spatial-dependent learning was correlated with the traffic flow transmission.

### 3.3. Learning Multi-Scale Time Dependency Based on LSTM-GRU Network

The third part of the model is the LSTM-GRU module. After completing the scale segmentation and spatial dependence learning on the time-series data, we constructed the LSTM-GRU composite network to learn the multi-scale time dependence. We used this module to train and forecast the traffic flow data. 

The module solves the problem of classical time-series data analysis. The most widely used and most effective method for processing time-series data is to use the LSTM or another RNN variant neural network for training. After solving the problem of non-RNN-like networks not utilizing the time dependency between the historical data, the remaining key to improving the prediction performance is the training decision combined with the actual situation. The time dependence has a strong periodicity, but the traffic timing data are not strictly periodic. Due to the peak periods and traffic accidents, the traffic state timing will oscillate, and the traffic flow prediction will be affected by a long-term periodicity and instability. Therefore, the predictive model is required to capture periodic or aperiodic changes from the complex traffic states and detect its end in time to achieve a rapid response to dynamic changes between traffic networks.

**Figure 4:**
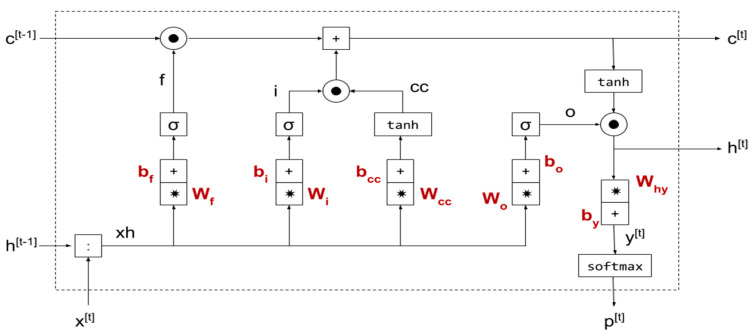
Diagram of the LSTM-GRU model (“*” in the figure is the symbol of multiplication).

The LSTM network is an improvement in the gradient explosion or gradient disappearance problem common to traditional RNN networks. The gating mechanism allows for a long-term sustainable gradient flow. The GRU network combines the two gating mechanisms of LSTM into one, which simplifies the structure and improves the operation speed under the premise of a low precision loss. Based on the time-series data scale segmentation of the MS-LSTM module, the LSTM network is selected for a high-precision prediction of hotspots in urban arteries that the fine-grained Link scale focuses on in the LZ-GCN module. For areas where the coarse-grained Zone scale focuses on less traffic flow data, GRU is selected to perform a full coverage prediction of the entire network. Combining the LSTM and GRU networks to form a time-series data training network not only improves the output accuracy of the time-series data for long time spans and large coverage areas, but it does not lead to an excessive increase in the time cost of model training and prediction. Our LSTM-GRU time-dependent learning module is shown in Figure 4.

## 4. Experiments

### 4.1. Data Description

In the experiments presented in this paper, we used a real-world dataset from New York City to evaluate the performance of the GLSNN model. The dataset is mainly divided into two parts: the detector dataset and the taxi dataset [20]. All the data were downloaded from the official website to ensure their authenticity and reliability. The urban artery traffic flow prediction used traffic camera data from the New York City Department of Transportation (NYC-DOT). The cameras are mainly distributed on the main roads of New York City and are relatively dense. The taxi dataset contains vehicle data covering all taxi travel trajectories throughout the entire New York City road network, issued by the New York City Taxi and Limousine Commission (NYC-TLC). The dataset was used to forecast the traffic situation of the whole city under the assumption that regional road traffic conditions are related to each other. We used these two time-series datasets as the experimental data for the training and prediction of the model.

(1) Taxi travel data

A cabs’ trajectory is one of the most commonly used data sets for predicting the traffic flow. Cabs trajectory datasets are being collected when taxi drivers are routing around the cities, so cabs trajectory data can provide urban flows of the passengers. In addition, by analyzing the temporal trajectory data to obtain the speed of cabs at different moments, we can effectively reveal the traffic flow situation of the urban road networks.

This study selected the taxi travel dataset from January to December 2016, with a total of 13,453 main roads in New York City as the study area, and constructed an adjacency matrix between them to describe the spatial relationship between the roads, which can better reflect the traffic zoning of New York City while taking into account the characteristics of New York passenger trips, which is helpful in predicting the traffic conditions associated with the city-wide road network. The size of the matrix was 13,453 × 13,453. Each line represents a road, and the connection between the roads was represented by 0 or 1. After that, the speed characteristic matrix was processed from the original taxi data to describe the change in the traffic speed with the time at a certain time on the road. Each row represented a road, and the values of the columns represented the speed of traffic on the roads at different times.

(2) Detector speed data

Except cabs, there are also a large number of buses and private cars in the city road network. Thus, the total traffic situation does not exactly match with the speed of a cab. By comparison, detectors densely distributed on hot roads in the city are more direct and accurate for the detection of the traffic flow. We selected the detector speed dataset from January to December 2016, which included a total of 1509 detectors set by the New York City DOT on trunk roads. Based on the distance between the detectors, we constructed a spatial weight matrix of 1509 × 1509. Then, a feature matrix describing the speed change was obtained by processing at a sampling interval of 5 min, responding to the traffic flow forecasting in the arterial areas of the city with a high precision.

Considering the obvious seasonal changes in urban traffic flow [21], we divided the datasets into four parts. For each quarter, we used the data of the first two months for training and the third month for testing. Thus, the segment strategy has the potential to obtain a more accurate prediction on a relative short time span (for example, 3 months). 

### 4.2. Evaluation Metrics

The diversification of the prediction methods and the multi-sourced input data make the establishment of more uniform and appropriate evaluation indicators a complex and challenging task. In order to evaluate the performance of the GLSNN prediction model, we synthesized the mainstream evaluation methods and reviewed the related literature. The following three metrics were selected to evaluate the model’s performance [17]: root means square error (RMSE), mean absolute error (MAE), and mean absolute percentage error (MAPE).

(1) $$\mathrm{RMSE}=\sqrt{\frac{1}{n}\overset{n}{\underset{i=1}{\sum }}{\left(Y_{t}-{\hat{Y}}_{t}\right)}^{2}}$$

(2) $$\mathrm{MAE}=\frac{1}{n}\overset{n}{\underset{i=1}{\sum }}\left|Y_{t}-{\hat{Y}}_{t}\right|$$

(3) $$\mathrm{MAPE}=\frac{1}{n}\overset{n}{\underset{i=1}{\sum }}\left|\frac{Y_{t}-{\hat{Y}}_{t}}{Y_{t}}\right|*100\%$$

note: the real traffic flow data are $Y_{t}$, and the predicted traffic flow data are ${\hat{Y}}_{t}$.

### 4.3. Experiment Settings

We used year-round detector data and taxi data to complete the forecasting task. These detectors are mainly distributed on the main roads with hotspots. The denser distribution and the sampling interval of 5 min allowed us to easily obtain the traffic flow changes during the peak period of the urban arterial region. We used the actual travel distance between the detectors to calculate the inverse distance weights, thus obtaining a fine-grained and highly correlated spatiotemporal feature matrix of the detectors. For the taxi travel data, we adopted a different approach to data processing. Under the premise of fully considering the characteristics of different types of taxis, the taxi area’s capability to summarize the traffic conditions was taken into account. Reverse fitting was conducted on the OD matrix of the trip through the Google Directions API to overlay it with the detector data and map the results to the road network.

The experiment environment consisted of an i9-9900K CPU, an NVIDIA RTX 2080 Ti graphics card, and 16 GB of memory, using TensorFlow for training. The main hyperparameters in the model were the learning rate, batch size, epoch size, number of iterations, and number of hidden layers. We determined the most appropriate settings for each parameter after repeatedly testing them. When the number of hidden layers in the detector dataset was set to 128 and the number of hidden layers in the taxi dataset was set to 64, the prediction accuracy of the model was relatively optimal. In the MS-LSTM module, we specially processed its regularization parameters. For the learning rate setting, we gave priority to avoiding an over-fitting. In order to reduce the learning rate, the number of trees and the time cost of the training were appropriately increased, thereby reducing the weight of a single tree to avoid over-fitting. The number of trees in the final model tree structure was 4000, the learning rate was set to 0.005, and the input batch size was set to 64.

### 4.4. Experiment and Results

From the perspective of multi-scale input–output and spatiotemporal dependence, a comparative analysis with several existing classical algorithms such as ARIMA, SVM, SAE, GRU, and LSTM-GCN was conducted, and the impact of the time dependence and spatial dependence on the prediction of the traffic flow was verified by conducting simulation experiments from both different time periods and different regions to validate the model in terms of a time dependence and spatial dependence. 

#### 4.4.1. Prediction of Complex Traffic Flow in Different Time Periods

**Figure 5:**
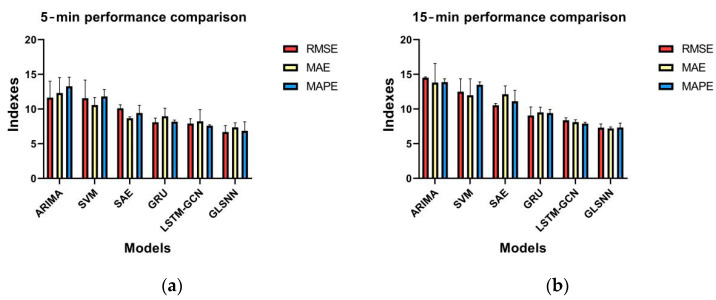

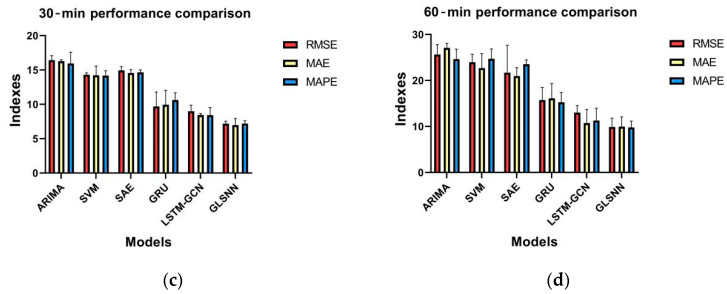
Comparison of model performance with prediction horizons of (**a**) 5, (**b**) 15, (**c**) 30, and (**d**) 60 min.

When implementing the time-dependent modeling, we first performed the scale segmentation of the complex traffic time-series data, which were then input into the time prediction model. As can be seen in Figure 5, the GLSNN model achieves a better prediction accuracy than other baseline methods, with an average error evaluation index being reduced by approximately 40% in the forecasting tasks with predicted steps of 5, 15, 30, and 60 min.

**Figure 6:**
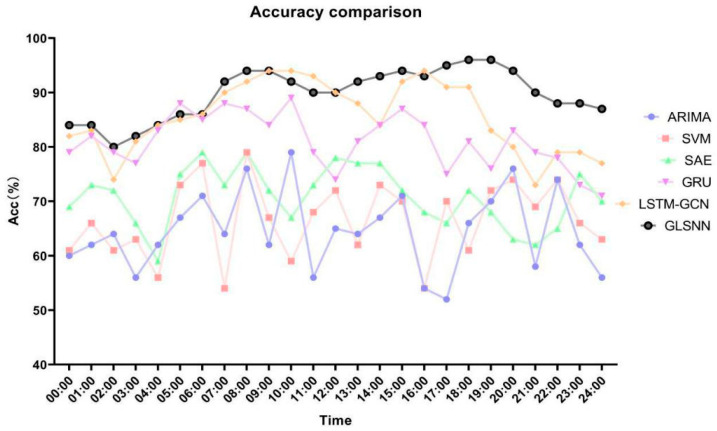
Prediction accuracy comparison. The Acc indicates the accuracy of the model’s prediction results.

The advantages brought by a scale segmentation for the time-series data inputs over a longer time span are also reflected in the superiority of the results of different time-period test sets (Figure 6). Figure 6 presents the model’s prediction accuracy on the traffic flow with a one-hour time interval in a day. In order to reduce the influence of stochastic factors and uncertainty, we did not select a typical day to analyze the 24 h traffic flow situation, but the average result of each hour of every day in the full year of 2016. A matrix of hour classes relative to the entire sample of 24 h in a year was constructed, and the hourly classes of the samples were obtained by combining the results of the tests related to the 5 min steps. From Figure 6, we can see that the GLSNN model shows an excellent prediction performance compared with the other algorithms. It has achieved a better prediction accuracy in most time periods throughout the day, with an average of the prediction accuracy reaching approximately 90%, and the fluctuation range of the prediction accuracy in different time periods is small. Even in the morning and evening peak hours and other periods of great change, it can keenly capture the changing pattern of the traffic flow for a high-precision prediction with more robustness.

#### 4.4.2. Traffic Flow Prediction in Different Urban Areas

At the beginning of the design of the GLSNN model, it strived to alleviate the influence of the interference of popular areas or the sparsity of unpopular areas in the data. In order to verify this feature, we selected two classic cases on the training dataset, namely a high-precision prediction in a hotspot in the city and a block prediction with a small traffic volume, training and visualizing the prediction results. 

**Figure 7:**
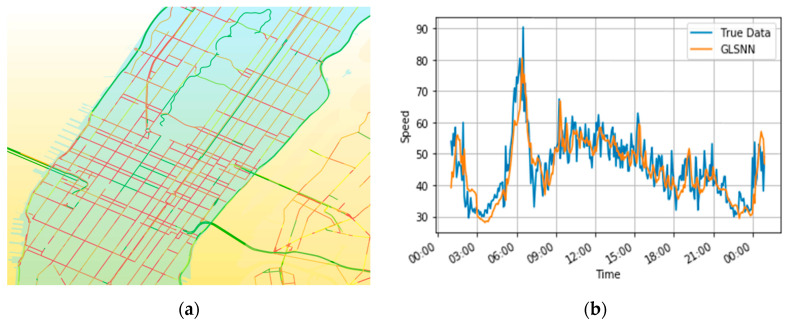
Visualization of traffic flow prediction results for city arteries: (**a**) the popular area of New York City; (**b**) the prediction results compared with real data.

In order to capture both the time-dependent and spatial-dependent characteristics, we employed the speed change in the neighborhood detector to predict the high traffic flow in urban aortic areas. Figure 7a shows the traffic flow pattern in the popular areas of New York City predicted by the GLSNN model, and Figure 7b represents the comparison of the traffic flow prediction results and the real values. Through inputting the traffic flow information of different spatial scales, the model can utilize the state diffusion and the transmission of the traffic flow to better perform a feedback adjustment. Therefore, when the traffic speed is oscillating, the model can still output more accurate prediction results, which reflects the robustness of the model. In Figure 7b, the hourly prediction performance comparison chart shows that the different rules for changes in the traffic flow, such as the commuting peak hours, work days, and weekends, can be better identified by the GLSNN model. For example, if the traffic speed oscillates greatly during the morning peak period of 5:00 am–8:00 am and the evening peak period of 6:00 pm–7:00 pm, the model can still output better prediction results. 

**Figure 8:**
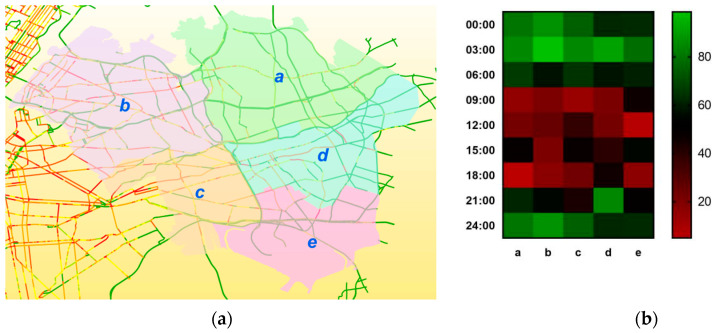
Visualization of network-wide traffic flow prediction results: (**a**) prediction results of different blocks divided by Zone with the clustering method; (**b**) the speed heatmap of each block.

Figure 8a shows the result of block prediction in the light traffic areas of New York City. Here, different block groups (e.g., a, b, c, d, and e, indicating neighborhood communities in light traffic areas of New York City, divided by Zone with the clustering method) were obtained by the Zone in the LZ-GCN module through clustering. Figure 8b is the speed heatmap of each block based on the real traffic flow data. Visually inspecting, the prediction results show a good consistency with the real value. For example, block b has the heaviest traffic flow among all the five blocks.

Whether in popular areas of the city (Figure 7) or in areas with fewer traffic volumes (Figure 8), with the advantage of a multi-spatial scale input, the GLSNN model can make full use of the complex road network topology combined with the spatial units of different scales to characterize the changes in the state of the traffic flow, so that the prediction results show a good consistency with the actual situation.

## 5. Discussion

In this paper, we constructed a GLSNN model by learning the temporal and spatial dependencies to improve the accuracy of an urban traffic flow prediction. Based on the simulation experiment results, the superiority of the GLSNN model in the spatiotemporal traffic prediction was proved. For the spatiotemporal prediction of the urban traffic flow, most previous models cannot capture time and space dependencies at the same time, which leads to changes in the traffic state not being captured and propagated [22,23]. Although a few studies simulated traffic flow by using both temporal and spatial information, most of them were only applied to small urban areas [1,5,15,22,23], which limited their practical value. 

In our model, the time-series data are not directly input into the model. We first perform a scale segmentation on the huge traffic flow data by the MS-LSTM module of the GLSNN model. The purpose of MS-LSTM is to use the ON-LSTM architecture to scale the data to integrate the tree structure into the LSTM network by learning the hierarchical structure information, so as to better use the time-series data to train the prediction model. Studies have shown that using hierarchical structure information contained in the time-series data is helpful to improve the accuracy of the prediction model [16]. In addition, compared with other methods, the GLSNN model is less sensitive to the field of view to be predicted, which contributes to a better prediction performance through training without considering how the prediction step size changes. Moreover, the GLSNN model can capture both multi-scale time and spatial dependences, and its capability to describe the spatiotemporal features of traffic is more powerful. Through the input of spatiotemporal features at different spatial scales, the complex topology structure of the urban road network can also be modeled more realistically. Aided by the multi-scale segmentation of the road networks, the GLSNN model is less affected by the interference of popular areas or the sparsity of unpopular areas in the data. As a result, different rules for changes in the traffic flow at different spatiotemporal scales can be better identified. The experiment results show that the average error of the evaluation index of our model was reduced by approximately 40% compared with the other baseline methods. However, it should be noted that a comparative analysis among different models for road traffic state prediction has always been a tough job, considering the complex road conditions and influence factors. Many proposed methods such as ARIMA, SVM, etc., have also been proved effective in predicting the state of the road traffic flow [21,24,25,26]. In this study, the comparison of existing classical models only considered the spatiotemporal combination and multiscale forecasting, and we only employed the accuracy as the indicator to evaluate the performance among different models, which may result in a biased estimation.

For the future improvement of the GLSNN model, some important factors should be incorporated. In recent years, with the advancement of intelligent transportation systems (ITS), Connected and Autonomous Vehicles (CAVs) have received widespread attention [27]. It can be foreseen that the heterogeneous traffic flow of human-driven vehicles (HV) and CAVs will appear on the same road [28]. Studies have shown that the differences in the car-following behaviors of HV drivers with diverse CAVs acceptances have much impact on the capacity and stability of the heterogeneous traffic flow [27,28]. Future studies should incorporate the potential impacts of CAVs on the mixed traffic flow prediction. Moreover, the impacts of non-traffic factors should also have been taken into account. Some studies have shown that non-traffic factors such as weather factors and spatiotemporal entropy during travel have a great influence on the prediction of the traffic flow [18]. A further improvement of the GLSNN model may incorporate the above non-traffic factors by means of correction coefficients for a comprehensive prediction [5].

## 6. Conclusions

In this paper, we propose a novel model of GLSNN for the prediction of the traffic flow. The GLSNN model can be used as a general framework for processing the structured time-series data. Different from the previous prediction methods, we have adopted a differentiated processing strategy. The experiment results based on the real traffic datasets validated the effectiveness of the GLSNN model on the large-scale prediction of an entire urban network and a high-precision prediction of urban arteries. The study highlighted the advantages of spatiotemporal-dependent learning in the performance evaluations and demonstrated the versatility of a multi-source data fusion in classic scene testing and improved the stability and anti-interference of the prediction model.

## Data Availability

Not applicable.
